# Obesity and Risk of Hypertension in Preadolescent Urban School Children: Insights from a Developing Country

**DOI:** 10.21203/rs.3.rs-4213965/v1

**Published:** 2024-04-12

**Authors:** Samina Akhtar, Shahid Khan, Namra Aziz, Muhammad Imran Magsi, Zainab Samad, Romaina Iqbal, Aysha Almas

**Affiliations:** Aga Khan University; Aga Khan University; Aga Khan University; Aga Khan University; Aga Khan University; Aga Khan University; Aga Khan University

**Keywords:** School going children, preadolescents, hypertension, systolic blood pressure, diastolic blood pressure, obesity, central obesity

## Abstract

**Background:**

Childhood obesity and hypertension are growing concerns globally, especially in developing countries. This study investigated the association between overall and central obesity at baseline, and prehypertension or hypertension at follow-up among preadolescent school children in Karachi, Pakistan.

**Methods:**

This is a sub study with cohort design embedded within a feasibility trial on School Health Education Program in Pakistan (SHEPP) in preadolescent aged 6–11 years, attending two private schools, were enrolled from 2017 to 2019. Hypertension or prehypertension at follow-up were the outcomes and obesity or central obesity at baseline were the exposure variables. Hypertension was defined as systolic blood pressure and/or diastolic blood pressure ≥ 95th percentile for age, sex, and height. Obesity was defined as body mass index for-age and sex ≥ 95th percentile, whereas central obesity was determined by waist circumference measurements ≥ 85th percentile of age, sex, and height specific cut-offs. Logistic regression analysis was used to calculate odds ratios (ORs) and 95% confidence intervals (CIs) to identify risk factors for hypertension and prehypertension.

**Results:**

Analysis was conducted for 908 participants, evenly distributed with 454 boys and 454 girls. Hypertension was observed in 19.8% of the preadolescents, with rates of 18.5% in boys and 21.0% in girls. Prehypertension was found in 16.8% of preadolescents, with 18% among boys and 16% among girls. Additionally, 12.8% of preadolescents were classified as obese and 29.8% had central obesity. Obesity at baseline was associated with hypertension (OR 8.7, 95% CI 3.5, 20.4) in the final model after adjusting for age, gender, physical activity, sedentary behavior, fruits, vegetable intake and hypertension at baseline. Central obesity at baseline also yielded high odds, with prehypertension (OR 1.9, 95% CI 1.4, 2.8) and hypertension (OR 2.7, 95% CI 1.9, 3.9) in the final model.

**Conclusion:**

This study highlights a concerning prevalence of hypertension and prehypertension among preadolescent school-going children. Obesity and central obesity at baseline emerged as significant predictive factors for hypertension within this cohort. The findings emphasize the urgency of implementing comprehensive school health education programs aimed at early detection and effective management of hypertension during childhood and adolescence in school settings.

## Background

Hypertension in children has become a public health concern globally (1). Prehypertension during childhood serves as an indicator for the onset of hypertension in adulthood. Hypertension in childhood has been associated with various end-organ complications, such as hypertensive retinopathy, kidney damage leading to microalbuminuria, and learning disabilities (2). Among the risk factors for hypertension, obesity stands out as a major contributor (3). Elevated Body Mass Index (BMI) in childhood and adolescence is linked to an increased risk of cardiovascular diseases in adulthood (4).

The prevalence of overweight (including obesity) among children and adolescents aged 5–19 has surged globally from 8% in 1990 to 20% in 2022 (5). This trend is particularly concerning in the South Asian population. According to a recent meta-analysis, included studies from 1994 to 2023, the prevalence of obesity and overweight among South Asian children and adolescents is 19.3% (6).

Thirteen percent of children in Karachi, Pakistan were found to have obesity, as indicated by higher BMI, while 21% have central obesity (7). Waist circumference (WC) is regarded as a better indicator of central adiposity and a reliable predictor of cardio-metabolic risk (8).

The risk of hypertension has been amplified by shifts in dietary patterns, including the increased consumption of junk food with high salt and saturated fatty acids, as well as insufficient intake of fruits and vegetables (9). A recent study on Chinese adolescents revealed that those consuming ≥ 3 servings of vegetables daily exhibited a lower risk of hypertension compared to those with daily vegetable consumption of < 1 serving (10). However, contrary findings were reported by Damasceno (11) and Mahfouz (12) who did not observe an inverse association between vegetable consumption and hypertension. Additionally, it’s noteworthy that the impact of vegetable intake on blood pressure has often been assessed in conjunction with fruit consumption (13).

Reduced physical activity and a sedentary lifestyle among children is contributing to the prevalence of obesity and hypertension (14). World Health Organization (WHO) recommends children between the ages of 5 and 17 years should participate in a minimum of 60 minutes of moderate-to-vigorous-intensity physical activity (MVPA) daily (15). However, in lower-middle-income countries (LMICs) such as Pakistan, only 7% girls and 30% boys are meeting the recommended level of physical activity (16). A study conducted in affluent schools in Karachi revealed that lifestyles, influenced by factors such as attending tuition classes, excessive television viewing, spending time on the internet, and engaging in indoor games like video games, contribute to this trend (17).

Previous studies have primarily focused on the association of obesity and hypertension, overlooking the holistic lifestyle patterns including eating behaviors unique to children. Moreover, data from urban school settings from LMIC like Pakistan are also limited. Therefore, the objective of this study was to elucidate the associations between obesity or central obesity at baseline and hypertension or prehypertension at 2-year follow-up. Additionally, to also report the association between various food groups and hypertension or prehypertension among preadolescent school children from Karachi, Pakistan.

## Methods

This is a sub study with cohort design embedded within a feasibility trial on School Health Education Program in Pakistan (SHEPP) (18). SHEPP was a parallel group feasibility intervention trial conducted from September 2017 to November 2019. SHEPP was a 10-month intervention targeted on children, parents, and teachers, with a primary focus on children. The intervention comprised of physical activity sessions and healthy heart teaching. Preadolescents with overall obesity and central obesity at baseline (2017) were followed for prehypertension and hypertension at follow up (2019).

The study was conducted in two schools located in Garden and Karimabad, Karachi which represent lower to middle-income classes. Both schools are situated within 10-km from the study center and were selected based on feasibility of logistics. Each school has approximately 2000 students, both boys and girls. Children from class 2, 3 and 4 (6–11 years) were eligible to be enrolled in the study. Students suffering from any physical disability were excluded from study but were allowed to attend the SHEPP session.

The outcome in the study was hypertension or prehypertension at follow-up. Hypertension was defined as systolic blood pressure (SBP) and/or diastolic blood pressure (DBP) lies at ≥ 95th percentile for age, sex, and height. Prehypertension was defined as SBP and/or DBP lying at < 95th ≥ 90th percentile. A child was considered normotensive if SBP, or DPB or both lies < 90th percentile (19).

Exposure was obesity or central obesity at baseline. BMI was computed by dividing an individual’s weight in kilograms by the square of their height in meters (kg/m^2^). The body weight status of each participant was categorized as follows: normal weight (BMI ≥ 5th percentile and < 85th percentile), overweight (BMI ≥ 85th percentile and < 95th percentile), and obesity (BMI ≥ 95th percentile) (1). Central obesity was indicated as WC value is ≥ 85th percentile according to international age, sex and height specific cut-offs of WC (20).

Other explanatory variables included dietary assessments, conducted using a 24-hour dietary recall on a typical weekday. The number of desserts, sugar sweetened beverages and readymade food intake were recorded. Additionally, intake of raw fruit (> 2 serving/day) and vegetables (> 3serving/day), protein (> 5 serving/day) dairy (> 3 serving/day) and grain (> 6 serving/day) were recorded (21).

### Data collection

Data was collected by SHEPP trained data collectors at baseline and at 10 months of intervention on data collection forms regarding physical activity, 24-h dietary recall and physical measurement of blood pressure (BP), WC, height, and weight.

The number of raw fruit and vegetables, number of sugar sweetened beverages and snacks/day were recorded. 7-day physical activity (PA) in minutes was assessed using the validated Youth Physical Activity Questionnaire modified for children (22). Standard measures were used to record BP by using Omron m5 monitors with a pediatric cuff (23). Weight was recorded to the nearest 0.1 kilograms (kg) using Tanita’s digital weight scales (24). Height was measured in centimeters (cm) by using Community-setting aluminum scale. Weight and height were recorded for each participant in light clothing and standing barefoot. WC was measured to the nearest 0.5 cm with an anthropometric non-elastic measuring tape, after normal expiration, at the top of the iliac crest (25).

### Data analysis

We utilized the ‘Pedbp’ macro package for R (https://dewittpe.shinyapps.io/pedbp/) to calculate gender, age, and height-specific blood pressure percentiles (26). Additionally, we employed the ‘quickZ_CDC’ (https://cpeg-gcep.shinyapps.io/quickZ_CDC/) and ‘WCz’ (https://cpeg-gcep.shinyapps.io/WCz_cpeg/) macro packages for R to determine BMI (gender, age, height and weight specific) and WC (gender, age and height specific) percentiles, respectively. STATA version 14.2 and R Studio version 4.3.0. were used for analysis. Mean (SD) was used to report quantitative variables and frequency and percentage for categorical variables. The independent sample t-test was used to compare quantitative variables (physical activity, BP, BMI, and WC) between two groups (boys and girls). The Chi-square test was used to compare qualitative variables between the two groups.

To examine the relationship between exposure (obesity or central obesity) and outcome (hypertension or prehypertension) logistic regression was employed to calculate odds ratios (OR) and corresponding 95% confidence intervals (95% CI), adjusting for covariates. In assessing the association of obesity and central obesity with hypertension, BMI below the 5th percentile and WC below 75th were used as the reference category. The 1st model explored the association between obesity and hypertension adjusting for age and gender. The 2nd model adjusted for age, gender, and physical activity. The 3rd model included adjustments for age, gender, physical activity, and sedentary behavior. In the final model, adjustments were made for age, gender, physical activity, sedentary behavior, fruit, vegetable intake and hypertension at baseline.

## Results

A total of 1280 children were assessed for eligibility in the SHEPP, and the consent forms and assent forms were distributed to 1191 eligible parents and their children. The study recruited 982/1191(82.5%) participants at baseline. Analysis was conducted for 908 participants, as data on blood pressure, weight, and height were missing for 74 participants at follow-up. There was an even distribution of 454 boys and 454 girls (Table 1). The participants had a mean (SD) age of 7.8 (1.0) years. Mean (SD) SBP in boys was 104.5 (10.8) mmHg, 101.9 (10.8) mmHg in girls (P < 0.00).

Hypertension occurred in 19.8% (n = 180) of the participants, with a rate of 18.5% (n = 84) in boys and 21.0% (n = 96) in girls (p 0.31). Prehypertension was observed in 16.8% (n = 153) of the participants, with 18% (n = 81) in boys and 16% (n = 72) in girls (p 0.42). Overall, according to age and gender specific BMI percentiles, 12.8% (n = 116) preadolescents were obese and 10% (n = 96) were overweight and 30% (n = 271) had central obesity at baseline

### Lifestyle factors -Diet and physical activity among preadolescents

Approximately 52% (n = 473) of participants consumed at least > 2 servings of fruits per day, while only 6% (n = 58) consumed > 3 servings of vegetables per day into their daily routine. Statistically significant gender differences were identified in the mean intake of readymade items (p = 0.005), whereas other dietary variables, including dessert, beverages, dietary protein, dairy, sand grain showed no substantial variations between boys and girls (Table 2).

**Figure 1:**
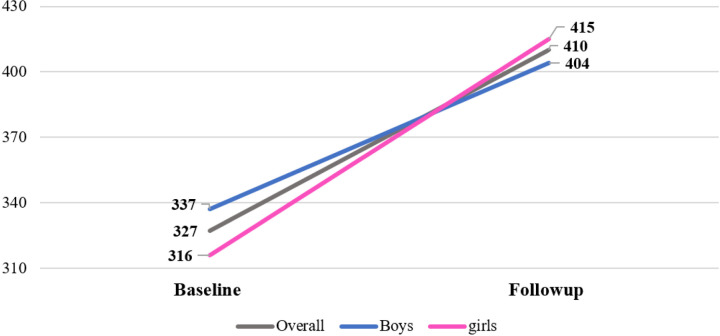
Mean 7-day physical activity levels at baseline and 10-month follow-up in preadolescent school children (n=908)

The average PA level of participants exhibited a notable increase from 327 to 410 per minute from baseline (2017) to after 10 months follow-up (2019). At the baseline, girls had a PA level of 316, which increased to 415, surpassing their counterparts. On the other hand, boys had a baseline PA level of 337, initially higher than the overall mean and at follow-up their PA level increased to 404. However, it decreased from the overall mean PA level of 410 (Fig. 1).

### Dietary intake and link to obesity and hypertension

**Figure 2:**
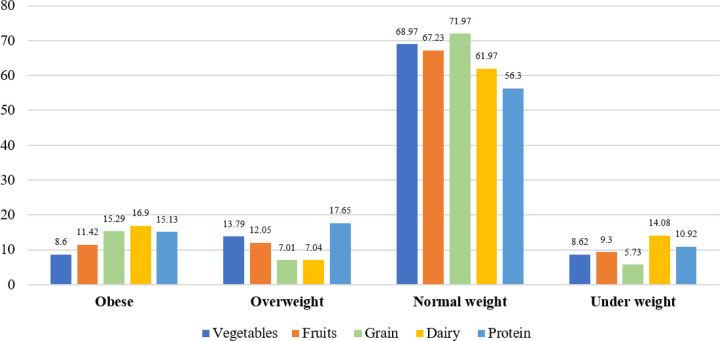
Dietary consumption across weight categories in preadolescent school children (n=908)

Among obese participants, the highest consumption was observed for dairy products (16.9%), followed by grains (15.29%) and protein (15.13%). Overweight participants showed high consumption of protein (17.65%). However, normal-weight children showed a balanced consumption pattern across all food groups (Fig. 2).

**Figure 3:**
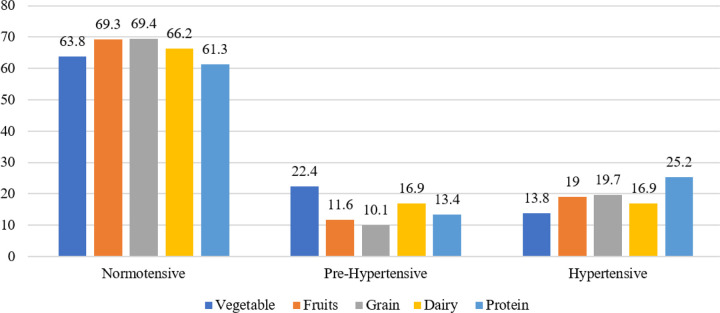
Dietary consumption across blood pressure categories in preadolescent school children (n=908)

Normotensive participants consumed relatively higher amounts of fruits (69.3%), grains (69.4%), and dairy products (66.2%) compared to their pre-hypertensive and hypertensive counterparts. Conversely, hypertensive participants demonstrated a notably higher consumption of protein (25.2%) (Fig. 3).

### Association of obesity with prehypertension and hypertension among preadolescents

The results in Table 3 revealed that obesity (BMI ≥ 95th percentile) at baseline is associated with prehypertension and hypertension at follow-up. The association remained in Model 2 (OR 5.5, 95% CI 2.2, 3.8) after controlling age, gender, and PA, and in Model 3 (OR 5.4, 95% CI 2.2, 13.7) after further adjustment for sedentary time. Notably, in the final model, the OR decreased to 4.6 (95% CI 1.8, 11.9) with additional adjustment for fruit, vegetable, and hypertension at baseline.

Similarly, higher estimates were seen for obesity with hypertension until Model 3 (OR 10.5, 95% CI 4.4, 24.7). However, in the final model, the OR decreased to 8.5 (95% CI 3.5, 20.4) after controlling for age, gender, PA, sedentary time, fruit, vegetable, and hypertension.

Likewise, baseline central obesity (WC percentile > 85) emerged as a predictor of both prehypertension and hypertension at follow-up. Central obesity not only yielded higher odds (Prehypertension: OR 1.9, 95% CI 1.4, 2.8; hypertension: OR 2.7, 95% CI 1.9, 3.9) at final Model but also demonstrated a statistically significant association, which was consistently observed across all models. The robustness of these associations was maintained even after adjusting for covariates.

### Association of dietary groups with prehypertension and hypertension among preadolescents

In exploring associations with different dietary groups, no consistent pattern emerged. The OR of 0.9 was consistently observed for dessert (95% CI 0.7, 1.3), beverages (95% CI 0.7, 1.1), readymade items (95% CI 0.9, 1.1), and grain (95% CI 0.6, 1.5) intake with prehypertension across nearly all models adjusting for covariates.

An intriguing finding emerged regarding dairy and vegetable intake, where the OR dropped from 1.3 (95% CI 0.7, 2.4) and 1.6 (95% CI 0.9, 3.0) for prehypertension to an OR of 0.9 (95% CI 0.5, 1.2) and 0.7 (95% CI 0.3, 1.5) for hypertension, respectively. Children consuming dietary protein exhibited an OR of 1.4 for both prehypertension and hypertension.

## Discussion

This study mainly evaluated the associations between obesity at baseline, and hypertension at follow-up among preadolescent school children in Karachi, Pakistan. In this paper we found that prevalence of hypertension and prehypertension was notably high, 19.8%, and 16.8%, respectively, with variations observed between boys and girls. A substantial proportion of preadolescents were identified as obese (12.8%), and central obesity was prevalent in 29.8% of the participants. After adjustments for covariates, including age, gender, PA, sedentary time, fruit, vegetable intake and hypertension at baseline, the study revealed an association between obesity and hypertension (OR 8.5, 95% CI 3.5, 20.4). Similarly, central obesity exhibited a statistically significant association with both hypertension (OR 2.7, 95% CI 1.9, 3.9) and prehypertension (OR 1.9, 95% CI 1.4, 2.8).

The existing body of literature consistently highlights a concerning upward trend in the prevalence of hypertension, particularly among younger age groups (27, 28). For instance, a study involving 22,224 students aged 10 to 17 from schools in the USA found that 16.3% met the criteria for prehypertension as per the new American Academy of Pediatrics (AAP) guidelines (29). Additionally, a study from Pakistan on children aged 5–14 years reported a prevalence of hypertension at 18.0%, with rates of 18.4% in boys and 17.6% in girls (30). These findings are consistent with our research. However, our study found a higher prevalence of prehypertension (16.8%) compared to a study conducted in Karachi, which reported a prevalence of 14.5% among children aged 8 to 12 years (1).

Genetic predispositions, lifestyle differences, and environmental factors unique to each study population could contribute to variations in the prevalence of hypertension (28). Also rising obesity among adolescents, increase sugar and beverage intake, reduced PA spaces could be contributory factor for obesity and hypertension (31).

Prehypertension and higher prevalence among girls could be attributed to the onset of puberty. Puberty represents a potentially crucial period in the development of prehypertension and may serve as an independent influencing factor. A research study focusing on school children aged between 7 to 12 years found that those who entered puberty or experienced early puberty exhibited increased odds of experiencing prehypertension. Notably, the study observed a more pronounced increase in blood pressure levels during puberty among girls and identified a stronger association between pubertal development and both hypertension and likelihood of prehypertension (32).

A substantial body of research emphasize the significant role of obesity as a key risk factor for hypertension, establishing a positive correlation between hypertension in children with high BMI (33). A systematic review including thirteen longitudinal studies up to June 2013 investigated the association between childhood obesity and adult morbidities. The findings suggests that overweight children are at a higher risk of developing diabetes and coronary heart disease in adulthood (34). A systematic review and meta-analysis, including 23 studies and 21 studies up to June 2015 respectively, provided additional insights. These analyses revealed that childhood obesity is significantly and positively associated with adult SBP and DBP (35). This highlights the importance of addressing childhood obesity as a preventive measure against hypertension and related cardiovascular complications in adulthood.

Van Emmerik et al., revealed that for every 10 kg increase in body weight, there was a corresponding increase of 3.0 mmHg in SBP and 2.3 mmHg in DBP (36). Our data analysis similarly indicated that obese children exhibited an OR of 8.5 for hypertension, aligning with findings from other studies that emphasized a strong association between high BMI and WC with hypertension in children (37–39).

The data in our study revealed that central obesity was associated with an increased risk of both hypertension (OR 2.7) and prehypertension (OR 1.9). An analysis of community-based data (n = 1,278) from the Control of Blood Pressure and Risk Attenuation (COBRA) trial in Karachi, focusing on children aged 5–14 years was conducted to explore the relationship between WC and BMI with the age-related increase in BP over a 2-year follow-up period. The findings revealed that increases in WC and BMI over time were linked to elevations in both SBP and DBP (30). In another study conducted in China, the OR for WC was reported as 3.75, demonstrating a significant association with the prevalence of hypertension among children aged 6–15 years (37).

The phenomenon of adiposity rebound in children within this age group may contribute to the high prevalence of prehypertension. Adiposity rebound refers to the natural increase in body fat that occurs after a period of decreasing adiposity, and it could be one of the contributing factors to the prehypertension identified in our research.

The present study has both strengths and limitations. A notable strength lies in the large sample size of preadolescent school children from lower to middle-income classes, Furthermore, the study employs a robust analytical approach, utilizing various statistical methods such as t-tests, chi-square tests, logistic regression, and specialized R packages.

Limitations of this study include, firstly, variations in criteria for determining hypertension and prehypertension in children and adolescents, including screening standards from the AAP, WHO, and National Heart, Lung, and Blood Institute. The different criteria might lead to discrepancies in directly comparing or generalizing the findings, as different standards may yield different prevalence rates of hypertension and prehypertension. Secondly, we did not measure Tanner stages as pubertal development may influence BP levels. Thirdly, the study was conducted in private schools within the city, which may not provide a comprehensive representation of government sector schools. Children in government institutions often come from relatively low-income families and may face challenges in maintaining a healthy lifestyle.

Future research endeavors should explore dietary patterns and consider additional risk factors such as puberty development and family history of obesity.

## Conclusion

The present study reveals a concerning prevalence of hypertension and prehypertension among preadolescent school-going children in urban Karachi. Notably, obesity and central obesity emerge as key predictive factors for hypertension in this cohort. This highlights the urgency of implementing comprehensive school health education programs for prevention early detection and management of hypertension in childhood and adolescence. Holistic strategies focusing on behavioral and lifestyle modifications are imperative. Preventing hypertension in adolescence could prevent or reduce the risk of hypertension and its associated complications in adult life.

## Figures and Tables

**Table 1:** Demographics, hypertension, and anthropometrics characteristics of preadolescent school children, strati ed by gender (n = 908)

Variables	Overall n (%)	Boys (n = 454) n (%)	Girls (n = 454) n (%)	pvalue
Mean age (SD) in years	7.8 (± 1.0)	7.8 (± 1.0)	7.7 (± 1.0)	0.14
Mean systolic blood pressure (SD) in mmHg at follow-up	103.2 (± 10.6)	104.5 (± 10.8)	101.9 (± 10.1)	< 0.00
Mean diastolic blood pressure (SD) in mmHg at follow-up	69.4 (± 14.1)	69.4 (±9.5)	69.4 (± 17.4)	0.94
Prehypertension at follow-up	153 (16.8)	81 (18.0)	72 (16.0)	0.42
Hypertension at follow-up	180 (19.8)	84 (18.5)	96 (21.1)	0.31
Mean weight (SD) in kilograms at baseline	26.1 (± 7.0)	26.1 (+ 7.0)	26.1 (± 7.0)	0.97
Mean BMI (SD) at baseline	16.6 (± 3.1)	16.6 (± 3.1)	16.7 (± 3.2)	0.43
**Overall obesity at baseline (BMI percentile** *)				
Under weight (< 5)	86 (9.8)	50 (11.0)	36 (7.9)	0.04
Normal weight (≥ 5 & < 85	610 (67.2)	297 (65.4)	313 (68.9)	
Overweight (≥ 85 & < 95)	96 (10.57)	40 (8.8)	56 (12.3)	
Obesity (≥ 95)	116 (12.8)	67 (14.8)	49 (10.8)	
Mean waist circumference (SD) in cm at baseline	63.2 (± 8.3)	62.6 (± 8.3)	63.7 (± 8.3)	0.04
**Waist circumference at baseline (percentile** **)				
No central obesity (< 85)	637 (70.1)	332 (73.1)	305 (67.2)	0.05
Central obesity (≥ 85)	271 (29.8)	122 (26.9)	149 (32.8)	
Mean physical activity (SD) in minutes at baseline	327.1 (± 139.8)	337.8 (± 135.4)	316.4 (± 143.4)	0.02
Mean sedentary (SD) time in minutes at baseline	6165.0 (± 440.1)	6204.0 (± 412.6)	6126.1 (± 463.1)	< 0.00

* Age and gender specific percentile

** Age, gender, and height specific percentile

**Table 2:** Twenty-four-hour dietary intake in preadolescent school children (n = 908)

Food groups*	Overall n (%)	Boys (n = 454) n (%)	Girls (n = 454) n (%)	p value
Mean intake of dessert (SD)	0.2 (± 0.6)	0.2 (± 0.6)	0.2 (± 0.6)	0.34
Mean intake of beverages (SD)	0.9 (+ 0.9)	0.9 (+ 0.9)	0.9 (+ 0.9)	0.39
Mean intake of readymade (SD)	1.8 (+ 2.1)	2.1 (+ 2.3)	1.7 (± 1.9)	0.005
Protein (> 5 serving/day)	119 (13.1)	59 (13.0)	60 (13.2)	0.92
Dairy (> 3 serving/day)	71 (7.8)	37 (8.1)	34 (7.5)	0.71
Grain (> 6 serving/day)	157 (17.3)	77 (16.9)	80 (17.6)	0.79
Fruits (> 2 serving/day)	473 (52.09)	265 (58.37)	208 (45.81)	<0.001
Vegetable (> 3serving/day)	58 (6.39)	26 (5.7)	32 (7.05)	0.41

* FRUIT AND VEGETABLE PROMOTION INITIATIVE / A MEETING REPORT / 25–27/08/03, World Health Organization.

**Table 3:** Association of overall obesity and central obesity with blood pressure in preadolescents school children (n = 908)

	Prehypertension				Hypertension			
	Model 1* OR (95% CI)	Model 2** OR (95% CI)	Model 3*** OR (95% CI)	Model 4**** OR (95% CI)	Model 1* OR (95% CI)	Model 2** OR (95% CI)	Model 3*** OR (95% CI)	Model 4**** OR (95% CI)
**Obesity (BMI percentile)**								
Under weight (< 5)	Ref							
Normal weight (≥ 5 & < 85)	2.4 (1.0–5.7)	2.4 (1.0–5.7)	2.4 (1.0–5.6)	2.3 (0.9–5.5)	1.9 (0.9–4.5)	2.0 (0.9–4.5)	2.0 (0.9–4.5)	1.9 (0.8–4.3)
Overweight (≥ 85 & < 95)	3.6 (1.4–9.4)	3.5 (1.3–9.3)	3.5 (1.3–9.2)	3.2 (1.2–8.7)	4.3 (1.8–10.7)	4.5 (1.8–11.1)	4.5 (1.8–11.1)	4.0 (1.6–10.0)
Obesity (>95)	5.5 (2.2–13.8)	5.5 (2.2–13.8)	5.4 (2.2–13.7)	4.6 (1.8–11.9)	10.2 (4.3–24.1)	10.5 (4.4–24.8)	10.5 (4.4–24.7)	8.5 (3.5–20.4)
**Waist circumference in percentile**								
No central obesity (< 85)	Ref							
Central obesity (≥ 85)	2.2 (1.5–3.1	2.2 (15–3.1)	2.2 (15–3.1)	1.9 (1.4–2.8)	3.0 (2.1–4.2)	3.1 (2.2–4.4)	3.1 (2.2–4.4)	2.7 (1.9–3.9)

* Adjusted for age & gender

** Adjusted for age, gender, PA

*** Adjusted for age, gender, PA, sedentary

**** Adjusted for age, gender, PA, sedentary, fruits, vegetable intake, hypertension

## Data Availability

The datasets used and/or analysed during the current study are available from the corresponding author on reasonable request.

## Abbreviations

AAP: American Academy of Pediatrics
BP: Blood Pressure
BMI: Body Mass Index
DBP: Diastolic Blood Pressure
MVPA: Moderate-to-Vigorous-intensity Physical Activity
SBP: Systolic Blood Pressure
SHEPP: School Health Education Program in Pakistan
LMICs: Low- and Middle-Income Countries
WC: Waist circumference
WHO: World Health Organization
